# Semiconductor nanomembranes: a platform for new properties via strain engineering

**DOI:** 10.1186/1556-276X-7-628

**Published:** 2012-11-15

**Authors:** Francesca Cavallo, Max G Lagally

**Affiliations:** 1 University of Wisconsin-Madison, Madison, WI, 53706, USA

**Keywords:** Semiconductor, Nanomembranes, Strain, Electronic properties

## Abstract

New phenomena arise in single-crystal semiconductors when these are fabricated in very thin sheets, with thickness at the nanometer scale. We review recent research on Si and Ge nanomembranes, including the use of elastic strain sharing, layer release, and transfer, that demonstrate new science and enable the fabrication of materials with unique properties. Strain engineering produces new strained forms of Si or Ge not possible in nature, new layered structures, defect-free SiGe sheets, and new electronic band structure and photonic properties. Through-membrane elastic interactions cause the double-sided ordering of epitaxially grown nanostressors on Si nanomembranes, resulting in a spatially and periodically varying strain field in the thin crystalline semiconductor sheet. The inherent influence of strain on the band structure creates band gap modulation, thereby creating effectively a single-element electronic superlattice. Conversely, large-enough externally applied strain can make Ge a direct-band gap semiconductor, giving promise for Group IV element light sources.

## Review

The evolution to miniaturization of electronic device structures has also increased applications through novel approaches and designs. A platform technology aiding this evolution that has recently seen rapid development is based on the use of thin crystalline semiconductor sheets of the order of 100 nm or less, called nanomembranes, as an alternative to bulk substrates [1-19]. These nanomembranes can be completely freestanding or tethered to a substrate, and they can be flat [4,20] or shaped into three-dimensional (3D) structures [21-23]. Conventional top-down patterning techniques are used in the fabrication of the device structures, and conventional growth techniques are used in the creation of layered structures [1-22]. The bottom-up (self-assembly) part of the fabrication comes about via strain engineering, as we will describe.

Crystalline nanomembranes (NMs) are distinguished from bulk materials most significantly by thinness, flexibility, nearness of two surfaces or interfaces, and the essential fact that in some part of their processing, nanomembranes are free of any constraint: they are released from a rigid handling substrate via removal of a sacrificial layer. Unique structural, electronic, and optical properties have been measured for these nanomembranes, both for flat and curled films [1-19,24-26]. NMs may be transferred to a large variety of hosts. This ability to transfer has been successfully used for the fabrication of hybrid or highly mismatched single-crystal multilayer stacks and for the development of bendable and stretchable electronics [2,4,18,20].

In addition to exploiting thinness and transferability for fabricating novel devices, one can take advantage of the mechanical compliance of freestanding NMs to establish a uniform or spatially varying strain field in the thin crystalline sheet [4,20,27-29], in some cases producing strain distributions that are not possible in the bulk [28]. Elastic strain sharing between a crystalline SiGe sheet sandwiched between two crystalline Si sheets completely unsupported by a solid allows the fabrication of tensilely strained SiNMs [4,20,28]. This method has been developed to create defect-free single crystals of SiGe, something not feasible with conventional approaches [30]. A spatially varying strain field has been established in Si nanoribbons by growing ‘local stressors’ (e.g., Ge or InAs quantum dots (QDs)) rather than a uniform stressor layer [27,29,31]. Additionally, one can use applied mechanical strain, as opposed to lattice mismatch-induced strain, to create new properties [32]. In all these cases, induced strain gives us control over the lattice constant and the symmetry of lattice expansion or contraction.

Producing strain in a material offers the possibility of tuning material properties. In particular, in semiconductors, electronic band structure and charge transport are the essential properties that control device behavior. Strain can modify band gaps and carrier mobility, both on a global and a local scale [29,33]. For this reason, strain engineering has significant implications for the development of a crystalline nanomembrane-based technology. Examples include the fabrication of high-performance and novel electron device structures as well as nanoscale photonic and thermoelectric devices [1-19,34-36]. In other materials, such as oxides, strain sensitively affects magnetic, ferroelectric, and pyroelectric behavior [37].

Ge and Si combine to make an ideal model system for strain engineering studies in thin sheets. Ge has a lattice constant that is 4% larger than that of Si. Even at submonolayer Ge coverages, strain has a significant impact on the structure of the Si (001) surface, via modified step structure and surface dislocation formation, features that have been quantified with scanning tunneling microscopy a number of years ago [38-41]. We focus here on the recent scientific developments related to Group IV semiconductor NMs that emphasize new materials and structures with new properties that cannot be fabricated or obtained in other ways.

### Creating silicon sheets with unique strain symmetries

Crystalline nanomembranes offer a powerful platform for using and tuning strain to create materials that have unique properties which are not achievable in bulk materials or with conventional processes. Nanomembranes, because of their thinness, enable elastic strain sharing, a process that introduces large amounts of strain and unique strain distributions in single-crystal materials, without the formation of extended defects. The reason is that the strain energy in a material increases as its thickness increases; in contrast to the bulk, at the same stress, a thin sheet will not contain sufficient strain energy to create dislocations or does not contain sufficient strain energy to fracture [42]. It is thus possible to make new strained materials using crystal symmetry as the driver [28].

The experimental demonstration is done with a trilayer Si(110)/Si_(1-*x*)_Ge_*x*_(110)/Si(110) nanomembrane, an elastically twofold symmetric system in which it is possible to transfer strain that is biaxially isotropic [28]. Tensilely strained Si(110) has emerged as an option for complementary metal oxide semiconductor devices because of its high carrier mobility [36,43]. Traditional methods to fabricate tensilely strained Si(001) rely on epitaxial growth of a Si layer on plastically relaxed SiGe(001) substrates. This process does produce strained Si(001), although with nonuniform strain and with roughness. It is not effective, however, for fabricating strained Si(110). For a given Ge concentration, the kinetic critical thickness for plastic relaxation is much lower in the (110) than in the (001) orientation. As a consequence, strain grading in SiGe(110) results in a threading dislocation density that is more than ten times higher in (110)- than in (001)-oriented relaxed SiGe substrates [44]. Furthermore, other strain relief mechanisms, i.e., roughening [45] and mosaic tilt [46], have been reported for strain relaxation in SiGe(110).

**Figure 1:**
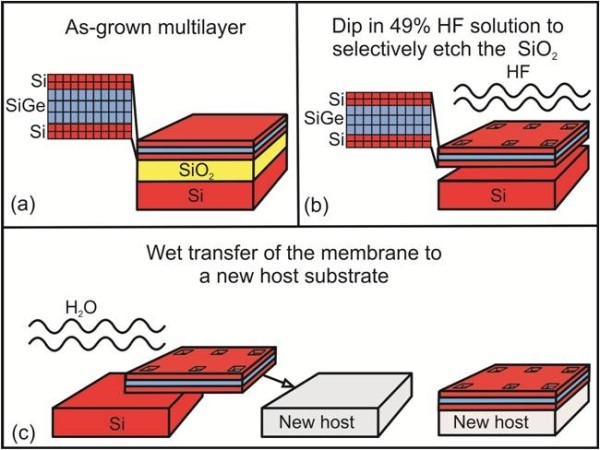
**Silicon nanomembrane release and transfer.** (**a**) As-grown Si/SiGe/Si trilayer. The distribution of the lattice constant across the trilayer is shown on the left-hand side; here, the SiGe is compressively strained and therefore tetragonally distorted, whereas the Si is unstrained. (**b**) The BOX layer is selectively removed with HF, releasing the membrane and allowing elastic relaxation via lateral expansion. As shown on the left-hand side, the released membrane has a less compressively strained SiGe layer sandwiched between two tensilely strained Si layers. An array of holes may be patterned on the unreleased membrane to enhance access of the etchant to the BOX. (**c**) Wet transfer of a released membrane from the original handling substrate to a new host surface.

One is, however, able to create tensilely strained Si(110) using nanomembrane strain sharing. In addition, this procedure will create a new strain symmetry. Fabrication of strained SiNMs (of any orientation) is schematically illustrated in Figure 1. The first step is the growth of a compressively strained SiGe layer (below its kinetic critical thickness for plastic relaxation) on the Si outer (template) layer of a silicon-on-insulator (SOI) substrate, followed by a top Si capping layer. The SiGe and top Si layers are pseudomorphically grown by chemical vapor deposition (CVD) or by molecular beam epitaxy. The distribution of the lattice constant across the trilayer is shown in the magnified image of the layer structure in Figure 1a,b. In the as-grown trilayer (Figure 1a), the SiGe is compressively strained and under tetragonal distortion, whereas the Si is unstrained, at the substrate lattice constant. Freestanding nanomembrane formation by removal of the underlying SiO_2_ is depicted schematically in Figure 1b. The release of the membrane from the SiO_2_ results in its overall lateral biaxial expansion, driven by the elastic strain relaxation of the SiGe layer. During this process, the compressive strain in the SiGe membrane partially transfers to tensile strain in the Si layers. Equal or nearly equal thicknesses of top and bottom Si layers are mandated to produce flat strain-balanced SiNMs after the release step. To enhance etchant access to the buried oxide (BOX), an array of holes may be patterned with photolithography and opened by reactive ion etching (RIE). Alternatively, strips of nanomembrane can be patterned, requiring no holes. For a typical set of holes (e.g., with 3 × 3 μm^2^ dimensions and a pitch of order 200 μm), only 0.06% of the membrane real estate is consumed by holes. SiNMs with sizes up to 400 × 400 μm^2^ can be rapidly released even without patterned holes. After BOX removal, the strain-relaxed crystalline trilayer membrane settles on the original Si substrate, where it weakly adheres. At this stage, it typically shows some wrinkles caused by the competition of the bonding of the NM to the underlying Si substrate and the continuing expansion as the NM is freed from the oxide (Figure 1b). Membrane transfer to a new host is achieved by either wet or dry transfer. In wet transfer, the released samples are agitated in water, generally with the addition of a solvent such as isopropyl alcohol, which allows the nanomembranes to separate from the handling wafer and float to the surface of the water. Membranes are then harvested from the solution by contact with a host substrate; they can be repositioned continuously without fracture until the water at the interface evaporates. The trilayer NMs, now fully elastically relaxed, bond to the new host (Figure 1c). This technique has so far been used to transfer membranes as large as 1 × 1 cm^2^ to a variety of substrates. The procedure is scalable to significantly larger dimensions and to almost any host substrate that is not rapidly dissolved in water. Alternatively, a dry printing method for transferring nanomembranes to any kind of new host can be utilized and is advantageous for some applications, notably for flexible electronics. Such methods are described in detail elsewhere [1,3-5,20]. Similar wet or dry transfer methods work for Ge NMs and those of other materials [18].

Continuum elasticity theory has been used to study the strain redistribution in a freestanding trilayer membrane with approaches based on energy minimization in the NM or force balance among the layers. For an ideal, elastically balanced Si/SiGe/Si trilayer with coherent interfaces, consideration of force balance allows the strain distribution between the SiGe layer and the Si layers to be calculated using

(1) $$\begin{array}{l} \varepsilon_{\text{SiGe}}\;=\;\varepsilon_{\text{m}}\frac{h_{\text{Si}}M_{\text{Si}}}{h_{S\text{iGe}}M_{\text{SiGe}}\;+\;h_{\text{Si}}M_{\text{Si}}}\\ \varepsilon_{\text{Si}}\;=\;-\varepsilon_{\text{m}}\frac{h_{\text{SiGe}}M_{\text{SiGe}}}{h_{\text{SiGe}}M_{\text{SiGe}}\;+\;h_{\text{Si}}M_{\text{Si}}}\text{,} \end{array}$$

where *ε*_m_ is the mismatch strain at the interface, and *ε*, *M*, and *h* are the layer strain, biaxial moduli, and thicknesses of the Si and SiGe layers [42]. Equation 1 shows that the use of high-Ge-content SiGe layers and ultrathin Si layers maximizes the strain in the two Si films. The upper limit to the thickness of the SiGe film is defined by the kinetic critical thickness for plastic relaxation of this layer during growth [47].

**Figure 2:**
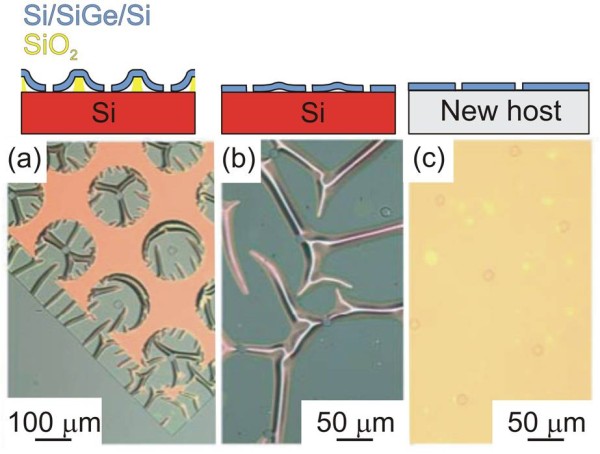
**Optical-microscope images of a (110) Si/Si**_**0.91**_**Ge**_**0.09**_**/Si NM.** The arrays of holes visible in the images are used to enhance etchant access. (**a**) Partially released membrane. (**b**) Membrane released in place. (**c**) Wet transfer to new Si host. The ripples visible in (b) smooth out when the membrane bonds to a new substrate surface. Schematic illustrations of the cross section of the multilayered structure at these stages of release and transfer are shown above the optical images. Images courtesy of Shelley Scott, ref [20].

Returning to the topic of this section, by utilizing elastic strain sharing [4,20] in Si/SiGe/Si multilayers, one can fabricate dislocation-free, biaxially tensilely strained (110)-oriented SiNMs, and furthermore, demonstrate a new strain symmetry that the material does not naturally possess [28]. Figure 2a,b,c shows optical-microscope images of (110)-oriented 12-nm Si/80-nm Si_0*.*91_Ge_0*.*09_/10-nm Si trilayer NMs at various stages of release and transfer. Figure 2a shows a snapshot of a partially released membrane, allowing observation of the etch front around the patterned holes and the edges of the membrane. The released parts relax by lateral expansion and form wrinkles because some parts of the released NM attach to the handling substrate before the NM is completely released. The continuing expansion of the sheet with some points already immobile causes the wrinkles. Figure 2b shows a completely released membrane after it has settled in place on its original growth substrate. The image in Figure 2c was obtained after transfer in fluid of a trilayer NM to a new Si host; both the membrane and the new host were chemically cleaned and H-terminated before transfer. The absence of wrinkling indicates that the membrane is smoothed during the float-off and transfer in fluid. Schematic diagrams of the cross section of the trilayer structures are shown above the optical images in Figure 2a,b,c to clarify the state of the NM during release and transfer. X-ray diffraction (XRD) measurements can be used, as shown in ref. [4] for strained (001) SiNMs, to confirm the structural coherence (e.g., lattice match, presence or absence of structural defects, SiGe layer compressively strained) of the Si/SiGe/Si trilayer NM after growth and after release and transfer of the NM to a new host. Surface morphology measurements, performed by atomic force microscopy (AFM), confirm the absence of any mechanism of plastic relaxation, i.e., nucleation of line and plane defects or roughening.

**Figure 3:**
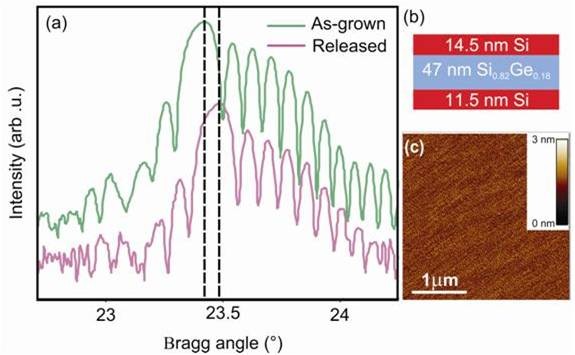
**Tensile strain in the (110)-oriented SiNM.** (**a**) XRD (*θ*/2*θ*) scans of the (220) reflection from a (110)-oriented 14.5-nm Si/47-nm Si_0*.*82_Ge_0*.*18_/11.5-nm Si heterostructure, before and after release in place. The SiGe peak and the interference fringes all shift by 0.064° to higher diffraction angle after release (indicated by the dashed lines). (**b**) Schematic diagram of the trilayer structure. (**c**) AFM image of the as-grown Si/SiGe/Si surface morphology. After ref [28].

The magnitude of the tensile strain in the (110) SiNM can be tuned by changing the Ge content in the alloy layer and the alloy layer thickness relative to the thickness of the SiNMs. A maximum tensile strain of 0.65 ± 0.01% in (110) SiNMs was calculated from XRD scans and confirmed by Equation 1. Figure 3a shows XRD *θ* − 2*θ* scans acquired around the (220) reflection of a 14.5-nm Si/47-nm Si_0*.*82_Ge_0*.*18_/11.5-nm Si trilayer membrane both before and after release. The single prominent peak visible in both scans originates from the SiGe layer. Because the Si layers are so thin, no prominent Si peak is visible. However, the presence of the strong interference fringes in the scans acquired before and after release indicates coherency at the interfaces.

After release, all peaks are shifted to higher diffraction angle, corresponding to a uniform in-plane expansion of the lattice. The uniform peak shifts and the interface fringes confirm that the SiGe layer has elastically relaxed during the release step. Transfer of the compressive strain in the SiGe film is determined by the relative thicknesses of the Si and SiGe layers. For the (110)-oriented trilayer schematically shown in Figure 3b, the in-plane biaxial tensile strain is *ε*_||_ = 0.49% in the Si layers after release [28]. The lack of significant surface roughening and crosshatch in these trilayer structures [28] indicates that there is no relaxation via 3D growth or misfit dislocation formation [48]. The surface roughness must be small so that the charge mobility enhancements resulting from strain are not negated by surface roughness scattering of charge carriers [49]. The absence of ridges in the Si capping layer excludes the presence of microtwins or mosaic tilt in the SiGe layer [28].

**Figure 4:**
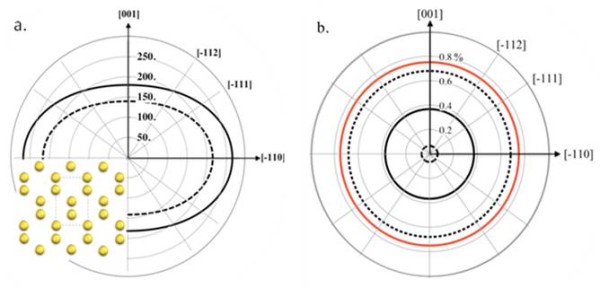
**Biaxial modulus and strain distribution in a Si(110) trilayer membrane.** (**a**) The modulus, *M*, for Si (solid line) and Ge (dashed line) as a function of in-plane direction on the (110) surface. The inset shows the atomic arrangement in the (110) plane. The crystallographic and elastic symmetry is twofold for the (110) surface orientation. (**b**) In-plane strain distributions in the balancing layers of trilayer NMs after elastic strain sharing for biaxially anisotropic balancing layers with a biaxially anisotropic stressor layer, corresponding to the Si(110) trilayer. Each curve represents a different thickness ratio between the outer balancing Si layers and the SiGe stressor layer: dotted line = 0.1, solid line = 1, and dashed line = 10. The mismatch strain in the system is shown as the red line; it corresponds to the strain in the stressor layer before strain sharing occurs and has the opposite sign as the strain transferred to the outer balancing layers. The radial distance to any curve is the magnitude of the strain in that direction. From ref [28].

The Si(110) system can explain the statement made above that one can create new strain symmetries using nanomembrane strain engineering. In a (110)-oriented trilayer heterostructure system, all of the layers have twofold elastic symmetry (Figure 4) but with an equibiaxial (isotropic) mismatch strain distribution. The mismatch strain is isotropic in-plane (Figure 4b) because the balancing layers and stressor layers have the same crystal structures, where the same in-plane orientations align during epitaxial growth (e.g., SiGe(110) on Si(110)). This means that the mismatch strain is defined in a similar way as a trilayer grown on (001)-oriented cubic materials. Although the biaxial modulus of Si(110) is biaxially anisotropic (Figure 4a), the similar crystal structures of Si and Ge result in similar elastic-constant anisotropies [28]. The implication is that strain transfer between (110)-oriented materials of similar crystalline structures remains biaxially isotropic (Figure 4b). These biaxially isotropic strain distributions in anisotropic materials are only enabled by elastic strain engineering in NMs.

### Mixed-crystal-orientation composite nanomembranes

The transfer of (110) SiNMs (even unstrained) to a (001)-oriented Si template and subsequent overgrowth can be used to fabricate mixed-crystal-orientation templates [50] (Scott SA et al., unpublished). This architecture may allow the fabrication, in close proximity to each other, of p- and n-channel devices on Si(110) (high hole mobility) and Si(001) (high electron mobility) regions, respectively, with the benefit of reducing the current drive imbalance between p-type metal oxide semiconductor (PMOS) and n-type metal oxide semiconductor (NMOS) devices [46]. Additionally, the ability to transfer a dislocation-free strained Si(110) nanomembrane to Si(001) promises hole mobility enhancements of up to 75% compared to the (001) universal mobility [43]. Furthermore, rotating the strained (110) membrane relative to its (001) host during transfer offers a concrete possibility of optimizing channel direction in n- and p-type devices.

**Figure 5:**
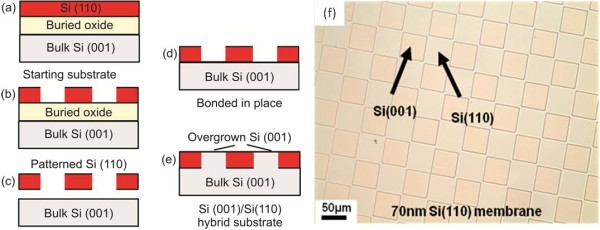
**Fabrication sequence of mixed-crystal-orientation nanomembrane via membrane transfer and overgrowth, schematically illustrated in cross section.** (**a**) The original SOI (110) substrate, (**b**) after lithography and RIE, (**c**) after removal of the buried oxide in HF, (**d**) after transfer and bonding to a bulk Si (001) substrate, and (**e**) after CVD growth of Si. The much faster growth rate of Si on Si(001) relative to that on Si(110) allows the holes to fill. The image at the right is after step (d). (**f**) Top-view optical image of a sample including (001)- and (110)-oriented regions.

**Figure 6:**
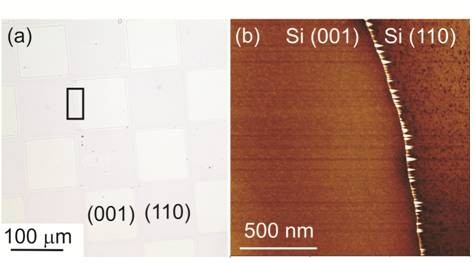
**Mixed-crystal-orientation nanomembrane after overgrowth.** (**a**) Optical-microscope image of a patterned 70-nm Si(110) membrane bonded on a Si(001) substrate after growth to fill in the holes in the Si(110) membrane. (**b**) AFM image including an interface between the two different orientations. From ref [50].

A mixed-crystal-orientation material in flexible-membrane form fabricated using SiNM transfer and overgrowth is schematically shown in Figure 5. SiNM fabrication begins with (110) SOI. The Si(110) template layer is patterned with an array of holes using standard photolithography and RIE and removed from its handle substrate via selective etching of the BOX layer in hydrofluoric acid (HF). This procedure creates a temporarily freestanding (110) SiNM, which is then transferred and bonded at 500*°*C to a Si(001) substrate. The holes define the regions where the Si(001) crystal planes are exposed through the membrane. Deposition of Si on the structure, using CVD with SiH_4_ precursor gas at a substrate temperature of 580*°*C, fills the holes because the Si growth rate is about ten times faster on the (001) orientation than on the (110) orientation at 580°C. Hence, fabrication of a flat mesh of Si(001) and Si(110) regions is achieved. Figure 6a is an optical-microscope image of a 70-nm (110) SiNM bonded to Si(001) and subsequently overgrown. The growth front is fairly planar, as indicated by the weak color contrast between the Si(001) and Si(110) areas in Figure 6a. Figure 6b shows an AFM scan across the intersection of the two different orientations, revealing that a surprisingly narrow line of defects exists along the boundary region between the two interfaces. The effect of such boundaries on electrical transport is so far not known (Scott SA et al., unpublished), but in applications in which these regions would be used separately, as in PMOS and NMOS devices in close proximity, this region is of no concern as it would typically be removed during shallow trench isolation during device fabrication.

### Mechano-electronic superlattice in a Si nanomembrane

It has been known for many years that the growth of Ge or Ge-rich SiGe on bulk Si(001) creates 3D nanocrystals (‘huts’ or ‘domes’, also called quantum dots) [51,52] that act as local stressors. They have random positions on the surface; the positional order can only be improved with the growth of multiple layers that act to self-organize the nanostressor arrangement [53]. On freestanding Si nanomembranes, growth also leads to the formation of nanostressors but with the distinct difference that the nanostressors self-organize already in a single layer if growth occurs on both sides of the NM. That is possible using CVD. Via through-membrane elastic interactions, the local strain created by a nanostressor provides a feedback for self-organization of the QDs, something that does not occur on bulk substrates. Strain sharing between the QDs and the compliant SiNM creates very small regions of high local strain in the membrane [29]. As a result, the SiNM undergoes distortion, forming into a slightly wavy sheet, with alternate regions of high tensile and compressive strains.

**Figure 7:**
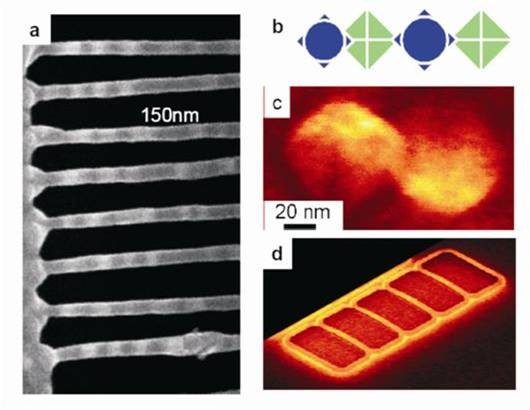
**Ordered Ge nanostressors on SiNMs cut into ribbons.** (**a**) Scanning electron micrograph of rows of pure Ge QDs on approximately 20-nm thick, approximately 80-nm wide Si(001) nanoribbons cut along [110] and tethered on both ends. The dots are clearly ordered and separated by approximately 150 nm on each surface. (**b**) Schematic diagram of dot ordering on the two surfaces of the ribbon. (**c**) Scanning electron micrograph of the relationship between nucleation on the top and on the bottom surfaces of a ribbon; 8-nm high, 80-nm base width Ge QDs on the top surface (left) and on the bottom surface (right). The Si ribbon is sufficiently thin so that a SEM images both sides simultaneously. When the electron beam is incident on the sample at a large angle with respect to the surface normal, the geometric projection of a nanocrystal on top (left) and bottom (right) surfaces of a Si membrane is different, and the two types of QDs can be distinguished. (**d**) SEM micrograph of a set of nanoribbons showing global bending caused by the average strain of the QDs. From ref [29].

Ordered growth of nanostressors has been observed on freestanding NMs in ribbon form with a 50- to 250-nm width, fabricated from SOI with 5- to 50-nm-thick Si templates [29,54]. Ge or a Ge-rich SiGe alloy is deposited simultaneously on both sides of these freestanding nanoribbons using CVD. Because CVD involves the vapor phase transport of precursor molecules, deposition is possible at all locations accessible to the growth gases (in contrast to molecular beam epitaxy, which is directional). The feedback mechanism briefly described above and explained in greater detail elsewhere [29,54-56] leads to a spatially periodic arrangement of pyramid-shaped QDs, alternating on the top and bottom surfaces of the SiNM. Consequently, a periodically varying strain is established along a thin single-crystal Si ribbon. This periodic strain in the Si membrane in turn produces a modulation in the electronic band structure that extends through the thickness of the membrane [29,57]. The modulated band structure therefore creates an electronic superlattice without a need for compositional modulation, the conventional method for forming electronic heterostructures. Figure 7a illustrates structures produced by depositing Ge or SiGe on both sides of freestanding nanoribbons formed from ultrathin (001) SOI. Growth on nanoribbons produces one or more lines of QDs, depending on the width of the ribbon, on both the top and the bottom surfaces of the ribbon. The QDs on the two surfaces of the ribbon assemble in a highly ordered lattice, as shown schematically in Figure 7b, which is square if two or more rows of dots form [54]. The QDs on each surface are aligned in rows along the *<*001*>* directions (i.e., the most compliant crystallographic direction in Si), but those on one surface are offset in the *<*110*>* direction from those on the other surface, as shown in Figure 7c, a scanning electron microscope (SEM) image of an 8-nm high pyramidal QD on the top surface (left) and one on the bottom surface (right). The alignment and offset are explained in terms of the anisotropic elastic constants of Si [54] and are independent of the crystallographic direction in which the ribbon is patterned on the SOI substrate. Figure 7d is a SEM image of four end-tethered nanoribbons illustrating the global response of the nanoribbons to the spatially varying strain field imparted to the NMs from the QDs. Once many QDs have formed on the ribbon, the overall effect of the QDs is like that of a continuous compressively strained film on both surfaces of the ribbon. For completely freestanding NMs, the strain can be relieved by lateral expansion [4,20]; in the particular case shown in Figure 7d, ribbons are end-tethered and thus forced to bow upward or downward.

The periodic strain field in the SiNM induces a periodic change in the Si band gap as a function of position along the ribbon. For this purpose, the local strain was modeled with a 2D finite element analysis of the elastic deformation and elastic energy resulting from two opposite-side QDs in one dimension, corresponding to the ribbon geometry with one line of dots on each side. The calculations [29] predict that the maximum tensile strain beneath an epitaxially grown Ge QD with a height of 8 nm and a base width of 80 nm is 1.62% for a 25-nm-thick ribbon and 1.89% for a 10-nm-thick ribbon. The resulting reduction of the band gap can be up to 250 meV for the thinnest ribbons, more than 20% of the bulk value of the band gap. The shift occurs almost completely in the conduction band [29]. The calculated band gap modulation in the SiNM due to the growth of nanostressors agrees with independently measured changes in the positions of bands for a uniformly biaxially strained SiNM [33].

A nanoribbon with a periodic change in the band offsets occurring essentially completely in the conduction band is equivalent to a one-dimensional (1D) periodic potential. One can therefore solve the Schrödinger equation to obtain the miniband structure in this 1D periodic potential. The results show that minibands with very small separations (i.e., minigaps) form within the potential well created by Ge nanostressors on Si but that only below 77 K would the thermal smearing be reduced sufficiently to make discrete minibands observable. A complete ‘phase diagram’ for possible band offsets and single-element electronic superlattices created by periodic strain as a function of nanostressor size, period, and NM thickness has been calculated [57].

**Figure 8:**
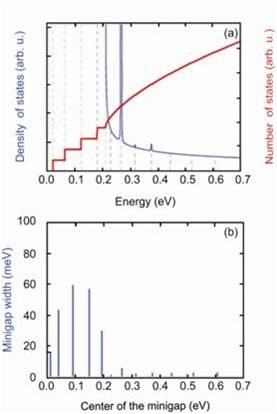
**Calculated total number of states (thick red line) and density of states (thin blue line).** For InAs QDs grown on a 25-nm-thick Si(001) NM. The reference energy level is the bottom of the quantum well. (**a**) For a potential amplitude *V*_0_ = 200 meV with period length of 11 nm, corresponding to 1.47% strain in 25-nm-thick Si. (**b**) Minigap width as a function of the energy in the middle of the minigap for the structure in (a). From ref [29].

With higher strain values, potentially obtainable with InAs quantum dots on SiNMs [31], it may be possible to observe discrete minibands in the potential well of the 1D strain superlattice at room temperature. The maximum tensile strain beneath a pure InAs pyramidal QD with a height of 6 nm and a base width of 11 nm pseudomorphically grown on a Si membrane is 1.47% for a 25-nm-thick ribbon and 2.68% for a 10-nm-thick ribbon. The resulting depression of the Si band gap is expected to be up to 350 meV, more than 30% of the bulk value of the band gap for the thinnest ribbons. Figure 8 shows the calculated density of states and total number of states for a 1D Si strain superlattice created by ordered, fully compliant, pseudomorphic InAs QDs. For a 25-nm-thick membrane, potential well depths reach 200 meV (Figure 8a), with minigaps ranging from 15 to 85 meV (Figure 8b). Only the first minigap is smaller than the thermal energy of approximately 26 meV at 300 K. It should therefore be possible to observe discrete minibands in the potential near room temperature if it were possible to grow InAs QDs that are not plastically relaxed but share their strain [31].

### Mechanical biaxial strain in Ge: making Ge a direct-band gap material

Mechanically straining freestanding NMs can transform Ge into a direct-band gap, efficient light-emitting material if sufficient strain can be induced. The work is based on the theoretical prediction that biaxial tensile strain in Ge has the effect of lowering the conduction-band edge at the direct (Γ) point relative to the L valley minima (which determine the fundamental, but indirect, gap at zero strain), while the overall band gap energy correspondingly decreases [58]. In the presence of electrical or optical pumping, a substantial population of electrons at the Γ minimum can therefore be established in sufficiently tensilely strained Ge, thereby increasing the light emission efficiency and enabling optical gain. If the strain exceeds 1.9%, the fundamental band gap even becomes direct.

**Figure 9:**
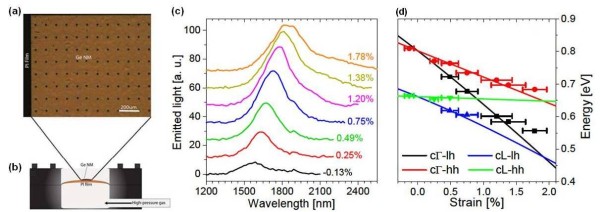
**Direct band gaps in tensilely strained Ge.** (**a**) Micrograph of a Ge NM bonded on a PI film. (**b**) Diagram showing the sample mount. (**c**) PL spectra of a 40-nm Ge NM at different strains. The increasing shoulder at higher wavelength reflects the lower-energy transition (black squares in (d)). (**d**) Symbols indicate the experimental emission energies obtained from the PL spectra of (c), plotted as a function of strain. Lines indicate the calculated Ge band gap energies versus strain. From ref [32]

The high levels of tensile strain required can only be obtained via mechanical deformation of freestanding Ge NMs [32]. Because of their nanoscale thicknesses, these nanostructures have much higher strain thresholds for plastic deformation and cracking compared to bulk Ge samples [42]. The Ge NMs are fabricated by releasing the top Ge layer of (001) Ge-on-insulator substrates, using a wet etch to dissolve the underlying buried oxide. The resulting freestanding NM is subsequently transferred and bonded to a flexible polyimide (PI) film [32]. A top-view micrograph of a Ge NM bonded on PI is shown in Figure 9a. The PI film is used to seal an otherwise rigid cavity that is then filled with high-pressure gas, as shown schematically in Figure 9b. With this arrangement, the film and the attached NM are biaxially stretched in a highly controllable fashion. The NM strain is measured as a function of the applied gas pressure via Raman spectroscopy, a technique that is extremely sensitive to strain in Si or Ge.

Figure 9c shows room-temperature photoluminescence (PL) spectra measured from a 40-nm-thick Ge NM at different strains below its threshold for plastic deformation [32]. The integrated luminescence is significantly enhanced with increasing strain. In Figure 9d, the solid lines show the calculated band gap energies between the Γ or L conduction-band minima and the heavy-hole or light-hole valence-band maxima as a function of strain. In general, all four transitions shown in this graph can contribute to the PL spectra, although depending on the strain, some of them may be nearly degenerate (or too weak) so that the corresponding emission peaks cannot be resolved. The calculations show that Ge has become direct band gap at an equibiaxial tensile strain of approximately 1.8%, i.e., the Γ valley of the conduction band has moved in energy to below the L valley. The direct-gap energy at this crossover position is approximately 0.47 eV.

## Conclusions

This brief review has summarized recent work on crystalline Group IV semiconductor nanomembranes. We have focused on work emphasizing novel science that results from using semiconductor sheets, i.e., structures in which one dimension is at the nanoscale while the other two are macroscopic. These sheets are single-crystal but extremely flexible. Because they are so thin, sheets can be highly strained. Combining growth and strain produces many new fundamental properties. Examples include the following: (1) Release of epitaxial layers of Si and SiGe from (110) SOI substrates induces elastic strain sharing among the layers, creating isotropically and biaxially strained Si(110), a strain symmetry that is not possible with bulk material. (2) The enhanced CVD growth of Si on Si(001) compared to Si(110) combined with bonding a meshed (110) membrane on top of a (001) substrate is used to create mixed-crystal-orientation surfaces consisting of uniform squares of Si(001) and Si(110). (3) 3D nanostressors that are a natural consequence of strained-layer growth produce local strain in freestanding NMs, and therefore local variations in the band gap of Si. The mechanical compliance of the NM allows the self-ordering of the nanostressors via through-membrane elastic interactions. The local strain can be made large enough to create band offsets in Si sufficiently large for miniband formation. Under some circumstances, the minigaps may be large enough to be observed at room temperature. (4) Using externally applied biaxial tensile strain, it is possible to change the band structure of Ge so that it becomes direct band gap. Thus, efficient light emission from Ge becomes possible.

These fundamental results suggest that Si and other semiconductor membranes are a disruptive technology for the development of novel device structures, allowing the integration of various functionalities (i.e., mechanical, optical, thermoelectric, and surface chemical) with high-performance electron devices.

## Competing interests

The authors declare that they have no competing interests.

## Authors’ contributions

FC and ML contributed equally in the preparation of this review. Both authors read and approved the final manuscript.
